# Mechanical Signatures of Tibiofemoral Cartilage Degeneration Identified by Unconfined Compression Testing: Implications for Early Osteoarthritis Risk in Athletes

**DOI:** 10.3390/medicina62040720

**Published:** 2026-04-09

**Authors:** Saida Benhmida, Ismail Dergaa, Halil İbrahim Ceylan, Nicola Luigi Bragazzi, Andrea de Giorgio, Hanene Boussi, Hedi Trabelsi

**Affiliations:** 1 Laboratory of Biophysics and Medical Technologies, Higher Institute of Medical Technologies of Tunis, University of Tunis El Manar, 9 Street, Doctor Zouheir Safi, Tunis 1006, Tunisia; benhmidasaida@gmail.com (S.B.); hanene.boussi@istmt.utm.tn (H.B.); heidi.trabelsi@gmail.com (H.T.); 2 High Institute of Sport and Physical Education of Ksar Said, University of Manouba, Manouba 2010, Tunisia; phd.dergaa@gmail.com; 3 Physical Activity Research Unit, Sport and Health (UR18JS01), National Observatory of Sports, Tunis 1003, Tunisia; 4 High Institute of Sport and Physical Education of El Kef, University of Jendouba, Jendouba 7100, Tunisia; 5 Physical Education and Sports Teaching Department, Faculty of Sports Sciences, Ataturk University, Erzurum 25240, Turkey; 6 Laboratory for Industrial and Applied Mathematics (LIAM), Department of Mathematics and Statistics, York University, Toronto, ON M3J 1P3, Canada; 7 Artificial Engineering s.r.l, 80121 Naples, Italy; andrea@degiorgio.info

**Keywords:** articular cartilage, biomechanical properties, energy absorption, nonlinear phenomenological viscoelastic model, osteoarthritis, sports injuries, tibiofemoral joint, unconfined compression, viscoelastic modeling, Young’s modulus

## Abstract

*Background and objectives*: Articular cartilage provides low-friction articulation across joint surfaces, distributes loads, and absorbs stress, all of which are crucial mechanical functions of joints. Changes in the mechanical characteristics of cartilage are among the first signs of degenerative joint disease, and they are especially important for athletes who are subjected to high-impact, high-magnitude loading on a regular basis. The objective of this study was to: (i) compare the mechanical characteristics of tibiofemoral cartilage in healthy and osteoarthritic conditions across medial and lateral anatomical compartments; and (ii) use nonlinear phenomenological viscoelastic modeling in conjunction with unconfined compression testing to characterize compartment-specific viscoelastic behavior. *Materials and Methods*: Forty-six human tibiofemoral cartilage samples were collected during knee surgeries and classified as healthy (*n* = 17) or osteoarthritic (*n* = 29) and as medial (*n* = 26) or lateral (*n* = 20). Quasi-static unconfined compression tests were performed at 1 mm/min to obtain stress–strain responses, Young’s modulus, maximum compressive stress, and energy absorption. Viscoelastic behavior was analyzed using a nonlinear phenomenological viscoelastic model. Appropriate parametric or non-parametric statistical tests and effect size measures were applied. *Results*: Osteoarthritic cartilage’s stiffness and energy absorption were significantly higher than those of healthy tissue (*p* < 0.05). Medial cartilage exhibited significantly greater stiffness and stress than lateral cartilage (*p* < 0.001). The nonlinear phenomenological viscoelastic model provided an excellent fit (R^2^ > 0.999). *Conclusions*: The mechanical profile of osteoarthritic tibiofemoral cartilage is characterized by pathological mechanical remodeling and increased stiffness. Greater mechanical susceptibility in the medial compartment supports the significance of cartilage biomechanical properties as sensitive indicators of early degeneration and osteoarthritis risk in athletic populations.

## 1. Introduction

Articular cartilage is a highly specialized connective tissue that covers diarthrodial joint surfaces and provides essential biomechanical functions, including load distribution, shock absorption, and low-friction articulation [1]. These properties arise from the unique composition of the extracellular matrix, predominantly collagen type II and proteoglycans, and from its biphasic structure, in which solid matrix components interact with interstitial fluid to generate nonlinear, time-dependent viscoelastic behavior [2]. This functional architecture enables cartilage to withstand substantial compressive forces over millions of loading cycles throughout life. However, the aneural and avascular nature of cartilage severely limits its regenerative capacity, making mechanical damage particularly problematic from both clinical and biomechanical perspectives [3]. Given that osteoarthritis affects more than 300 million people worldwide and represents a leading cause of disability, understanding cartilage mechanical behavior remains a major research priority [4].

Athletic populations engaged in high-impact sports experience accelerated cartilage degeneration compared with the general population. Football players are exposed to repetitive mechanical loading, rotational movements, and direct impacts that subject articular cartilage to stresses exceeding normal physiological ranges [5]. Epidemiological studies indicate that former professional football players develop knee osteoarthritis at younger ages and with higher prevalence than age-matched controls [6]. Sport-specific activities such as cutting, deceleration, and jumping generate peak joint forces reaching 6–8 times body weight, challenging the viscoelastic buffering capacity of cartilage [7]. Over time, these repetitive loads induce cumulative microstructural damage, including collagen network disruption, proteoglycan depletion, and surface fibrillation [8]. Clinically, cartilage lesions in athletes are frequently observed in weight-bearing regions of the medial tibiofemoral compartment, where mechanical stress concentrations are highest [9]. Notably, magnetic resonance imaging studies report cartilage abnormalities in up to 60% of elite football players even in the absence of symptoms, suggesting that mechanical damage precedes overt structural degeneration [10].

During osteoarthritic degeneration, cartilage mechanical properties undergo marked alterations. Healthy tissue exhibits strain-rate-dependent stiffness, nonlinear stress–strain behavior, and effective energy dissipation through viscoelastic mechanisms [11]. Degenerated cartilage is characterized by reduced proteoglycan content, collagen disorganization, and increased water content, which collectively modify its mechanical response [12]. Conflicting reports describe either increased or decreased apparent stiffness during disease progression, reflecting differences in disease stage, cartilage thickness, and subchondral bone involvement [13]. Importantly, regional variations in mechanical properties have been consistently reported, with medial compartments typically exhibiting greater stiffness and load-bearing capacity than lateral regions [14]. Parameters such as instantaneous modulus, relaxation behavior, and energy absorption capacity have emerged as sensitive indicators of early cartilage degeneration, often preceding morphological changes detectable by conventional imaging techniques [15].

Despite extensive research on cartilage biomechanics, important gaps remain regarding the relationship between mechanical property alterations and sports-related injury mechanisms. Most existing studies focus on osteoarthritic cartilage obtained from elderly patients undergoing total knee replacement, limiting their relevance to younger athletic populations experiencing early degenerative changes [16]. Furthermore, only a few studies have systematically compared compartment-specific mechanical behavior while integrating experimental testing with nonlinear viscoelastic modeling capable of capturing the strain-dependent stiffening observed under high-impact loading conditions [17].

Based on the identified research gaps, our study aimed to (i) characterize and compare mechanical properties of healthy and osteoarthritic articular cartilage across medial and lateral tibiofemoral compartments using unconfined compression testing, (ii) develop a nonlinear phenomenological viscoelastic model to describe the tissue’s mechanical response under quasi-static loading accurately, and (iii) interpret these biomechanical findings within the context of cartilage lesion mechanisms observed in high-impact athletic activities, particularly football.

## 2. Materials and Methods

### 2.1. Ethical Approval

All experimental procedures were approved by the La Rabta Hospital Ethics Committee and were conducted in accordance with the principles of the Declaration of Helsinki for human tissue research. Written informed consent was obtained from all donors or their legal representatives before tissue collection. Participants were adults aged 18 years or older without a history of inflammatory joint disease or previous knee surgery affecting the sampling sites.

### 2.2. Sample Collection and Classification

Human articular cartilage samples were harvested from the tibiofemoral joint during scheduled knee surgeries, including total knee arthroplasty procedures for osteoarthritic patients and tissue collection from post-mortem donors for healthy samples. Forty-six samples were collected from male and female donors aged 30 to 78 years. Cartilage specimens were extracted from two predefined anatomical regions of the tibial plateau: the medial compartment (M, *n* = 26) and the lateral compartment (L, *n* = 20). Samples were prepared with dimensions of approximately 10 mm in length, 10 mm in width, and 1–2 mm in thickness. The cutting axis was oriented perpendicular to the articular surface to obtain flat samples and ensure uniform stress distribution across the entire cartilage surface during compression testing. Cartilage thickness was measured using a digital caliper with 0.01 mm precision.

Specimens were classified according to health status: the healthy group (*n* = 17) included samples from donors without radiographic or clinical indicators of osteoarthritis (Kellgren-Lawrence grade 0–1), while the osteoarthritic group (*n* = 29) comprised samples from patients undergoing arthroplasty with Kellgren-Lawrence grade ≥ 3. Sample distribution across combined classifications was as follows: healthy-medial (*n* = 9), healthy-lateral (*n* = 8), osteoarthritic-medial (*n* = 17), and osteoarthritic-lateral (*n* = 12). Age stratification for subgroup analysis included three categories: less than 40 years, 40–60 years, and greater than 60 years.

Immediately following extraction, samples were rinsed in isotonic saline, stored in sterile containers in phosphate-buffered saline, and maintained at 4 °C until testing. All mechanical tests were performed within 48 h post-extraction to minimize post-mortem degradation.

### 2.3. Compression Testing

Compression tests were conducted using a LLOYD-EZ50 compression machine (Lloyd Instruments, Bognor Regis, UK) equipped with a 1 kN force sensor. The device was calibrated before each testing session in accordance with the manufacturer’s specifications. All tests were performed at room temperature (20 ± 1 °C) to maintain consistent testing conditions. Each sample underwent quasi-static unconfined compression testing with a preload of 0.01–0.05 N applied to ensure proper contact between the compression platens and cartilage surface. The compression rate was set to 1 mm/min, a standard rate for characterizing the viscoelastic behavior of soft biological tissues while minimizing fluid-flow effects.

Engineering stress (*σ*) was calculated using Formula (1), where *F* represents the measured force in Newtons, and *A* represents the initial cross-sectional area in square millimeters. Engineering strain (*ε*) was computed using Formula (2), where *ΔL* represents displacement, and *L*_0_ represents initial sample height. The elastic modulus (Young’s modulus) was calculated as the slope of the initial linear region of the stress–strain curve, as determined by linear regression, according to Formula (3). Energy density was computed for each sample as the area under the stress–strain curve, obtained by numerical integration using the trapezoidal method, as shown in Formula (4), where *ε*_max_ denotes the maximum strain achieved during testing. The resulting energy was expressed in kilojoules per cubic meter (kJ/m^3^).

(1) $$\sigma =\frac{F}{A}$$

(2) $$\varepsilon =\frac{\Delta L}{L0}$$

(3) $$E=\frac{\sigma }{\varepsilon }$$

(4) $$W=\int_{0}^{\varepsilon max}\sigma (\varepsilon )\;d\varepsilon \;\;$$

### 2.4. Viscoelastic Modeling

A nonlinear phenomenological viscoelastic model was employed to characterize the viscoelastic response of articular cartilage under quasi-static unconfined compression. This model was selected for its ability to reproduce the nonlinear stiffening behavior observed in biological soft tissues under monotonic loading. The total stress *σ*(*ε*) was expressed as the sum of an equilibrium elastic response and two nonlinear viscoelastic branches according to Formula (5), where *E∞* represents the equilibrium modulus, *E*_1_ and *E*_2_ represent the viscoelastic branch coefficients, and *α*_1_ and *α*_2_ represent the nonlinearity exponents governing the stiffening rate. This formulation captures the progressive increase in tissue stiffness with increasing strain, consistent with collagen fiber recruitment and matrix compaction during compression.

(5) $$\sigma \left(\varepsilon \right){=E}_{\infty }+E_{1}\varepsilon^{\alpha_{1}}+E_{2}\varepsilon^{\alpha_{2}}\;$$

In order to accurately depict the strain-dependent and time-dependent mechanical behavior of articular cartilage under quasi-static compression while balancing physiological relevance and model parsimony, the nonlinear phenomenological viscoelastic model was selected. Unlike linear viscoelastic formulations, this model structure explains the gradual stiffening observed at greater strain levels, which is a reflection of the intrinsic cartilage tissue mechanisms of matrix compaction and collagen fiber recruitment. The modeling of distinct relaxation processes working at different time scales is made possible by the use of two viscoelastic branches, which is consistent with the multicomponent character of the cartilage extracellular matrix. Because it provides sufficient flexibility to represent compartment-specific mechanical responses without over-parameterization, this model structure is perfect for comparing healthy and osteoarthritic cartilage.

The choice of the nonlinear phenomenological viscoelastic model was further supported by comparison with commonly used alternative approaches for cartilage mechanics. Classical linear viscoelastic models, such as Maxwell and Kelvin–Voigt formulations, are limited in their ability to reproduce the pronounced strain-dependent stiffening observed in articular cartilage. In contrast, more advanced biphasic and poroelastic models provide a detailed representation of fluid–solid interactions but require a large number of parameters and complex experimental calibration. The formulation used in this study provides an effective compromise between physiological relevance and model parsimony, allowing accurate representation of both instantaneous and time-dependent responses under quasi-static loading. Previous studies have demonstrated that cartilage exhibits highly nonlinear viscoelastic behavior associated with collagen network mechanics and matrix interactions [18,19]. Therefore, the selected model is well suited for capturing compartment-specific mechanical differences while maintaining computational robustness.

Parameter optimization was performed using nonlinear least squares regression implemented through the SciPy curve_fit function. Convergence was ensured using bounded initialization and physiologically reasonable starting points. Model performance was quantified using the coefficient of determination (*R*^2^). To evaluate model robustness, a global sensitivity analysis was conducted by perturbing each parameter independently by ±10% and recalculating the resulting *R*^2^. This procedure identified the parameters with the most significant influence on model stability for each anatomical compartment. All computational analyses were performed in Python version 3.10 within the Google Colab environment, using the NumPy, Pandas, and SciPy libraries.

To assess experimental repeatability, one representative cartilage sample was tested three times under identical conditions at the beginning of the experimental protocol. Calculated from the highest stress readings, the coefficient of variation (CV) was 18.2%, showing satisfactory repeatability. Because of the damaging nature of compression testing, the specimens’ limited thickness and size, and the scarcity of human biological material, not all samples underwent repeated testing. Strict experimental standardization, including systematic testing machine calibration, controlled ambient conditions, and a consistent loading process, was used throughout the study to guarantee measurement consistency. The variability of mechanical parameters within each group was quantified using standard deviations as an indirect indicator of measurement consistency. In addition, the stability of the viscoelastic model fitting, as reflected by consistently high coefficients of determination (*R*^2^ > 0.999), together with the sensitivity analysis, further supports the robustness and reproducibility of the experimental measurements.

### 2.5. Statistical Analysis

All statistical analyses were performed in Python using the SciPy and Pandas libraries. Data normality was assessed using the Shapiro–Wilk test. Depending on the distribution of each variable, the appropriate statistical tests were applied as follows. For normally distributed data with unequal variances, the Welch’s *t*-test was used to compare two groups (Healthy vs. Osteoarthritic and Lateral vs. Medial when applicable). For non-normally distributed variables, the Mann–Whitney U test was applied. An initial Kruskal–Wallis test served as a non-parametric omnibus test to explore global differences before performing pairwise analyses. Effect sizes were computed using Cohen’s d for parametric comparisons (Cohen’s d was computed as the difference between group means divided by the pooled standard deviation). For non-parametric comparisons, the rank-biserial correlation ($r_{rb}$) was derived from the Mann–Whitney U statistic as:

$$r_{rb}=1-\frac{2U}{\;n_{1}n_{2}}$$

where $n_{1}$ and $n_{2}$ are group sizes.

Effect sizes were interpreted according to conventional thresholds (small, moderate, and large effects). Small, moderate, and large effects were defined as 0.2, 0.5, and 0.8 for Cohen’s d, respectively. For rank-biserial correlation, thresholds of 0.1, 0.3, and 0.5 were considered small, moderate, and large effects. A significance threshold of *p* < 0.05 was used in all analyses.

### 2.6. Statistical Power Analysis

To confirm that the sample size was sufficient to detect significant differences in cartilage mechanical properties between groups, a post hoc statistical power analysis was performed based on the observed effect sizes of the primary outcomes. Power was calculated for independent two-tailed comparisons at a significance level of *α* = 0.05 using the achieved group sample sizes. Calculations were performed using the noncentral t-distribution framework (TTestIndPower, statsmodels, Python). Statistical power exceeded 80% for the principal comparisons, indicating adequate sensitivity to detect the observed effects.

However, it should be noted that post hoc power analysis is based on observed effect sizes and does not replace an a priori sample size estimation and should, therefore, be interpreted with caution.

### 2.7. AI Usage Declaration

In preparing this manuscript, the authors used the ChatGPT model 4 on 6 December 2025, to revise selected passages and verify grammar and academic English quality [20,21]. Following the use of the tool, the authors comprehensively reviewed and edited all content and assume full responsibility for the manuscript’s scientific accuracy and integrity.

## 3. Results

### 3.1. Stress–Strain Mechanical Response

**Figure 1:**
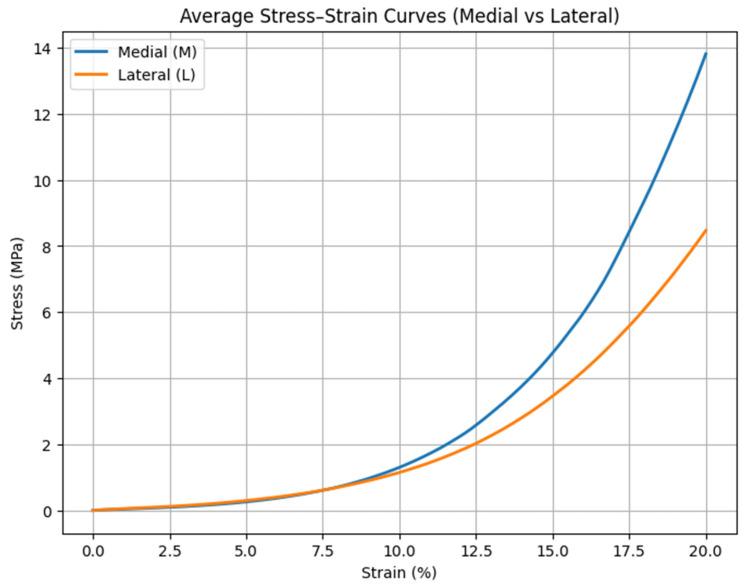
Mean stress–strain curves for Medial and Lateral compartments. Medial cartilage exhibits a stiffer response throughout the compression range, whereas lateral cartilage is more compliant at lower strain and stiffens sharply at higher deformation.

The averaged stress–strain curves revealed substantial differences in mechanical behavior between anatomical compartments and health status groups. Figure 1 demonstrates the mean stress–strain relationships for medial and lateral compartments. Across the strain range from 0 to 20%, the medial compartment exhibited higher stress values than the lateral compartment. The difference increased at higher strain levels, particularly beyond approximately 12–15% strain. The lateral compartment showed lower stress values at low strains, with a progressive increase at higher deformation levels.

**Figure 2:**
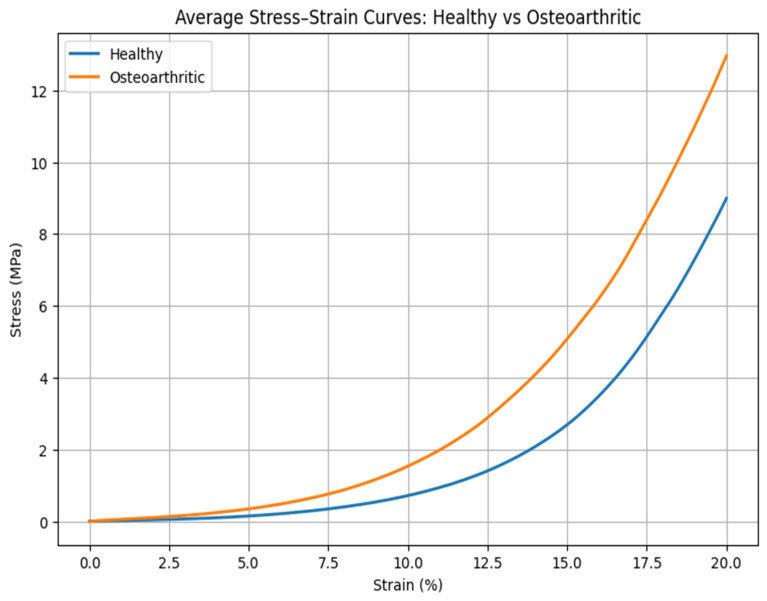
Representative stress–strain mean curves for Healthy and Osteoarthritic samples during quasi-static unconfined compression. Osteoarthritic cartilage exhibits altered mechanical response with greater deformation capacity (higher energy absorption) alongside structural changes that increase apparent stiffness at higher strain levels.

Figure 2 compares the stress–strain curves of healthy and osteoarthritic cartilage. Across the examined strain range, osteoarthritic cartilage displayed different stress–strain profiles compared with healthy tissue. At lower strain levels, osteoarthritic cartilage exhibited higher strain values for comparable stress levels, whereas at higher strain levels, it reached higher maximum stress values. These differences were more pronounced at higher deformation levels.

### 3.2. Energy Absorption Capacity

Table 1 presents the comparative analysis of energy absorption, Young’s modulus, and maximum stress across health status and anatomical location groups. Energy absorption differed significantly according to both health status and anatomical location. Osteoarthritic cartilage exhibited significantly higher energy absorption than healthy tissue. Similarly, the medial compartment demonstrated higher energy absorption than the lateral compartment.

**Figure 3:**
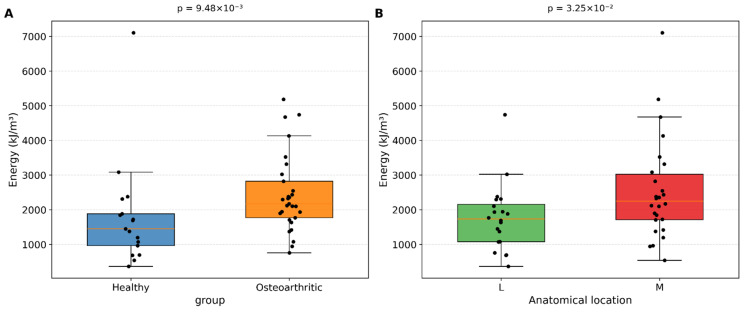
Comparative analysis of the energy absorption of articular cartilage across two key biomechanical determinants: (**A**) Boxplot comparing the energy between healthy and osteoarthritic samples, (**B**) Comparison of energy between the medial and lateral compartments.

Figure 3A illustrates the distribution of energy absorption between healthy and osteoarthritic samples. Figure 3B shows the variability observed in the medial and lateral compartments.

### 3.3. Young’s Modulus Distribution

Young’s modulus differed significantly according to both health status and anatomical location (Table 1). Osteoarthritic cartilage exhibited significantly greater stiffness compared with healthy tissue. Similarly, the medial compartment demonstrated higher stiffness than the lateral compartment.

**Figure 4:**
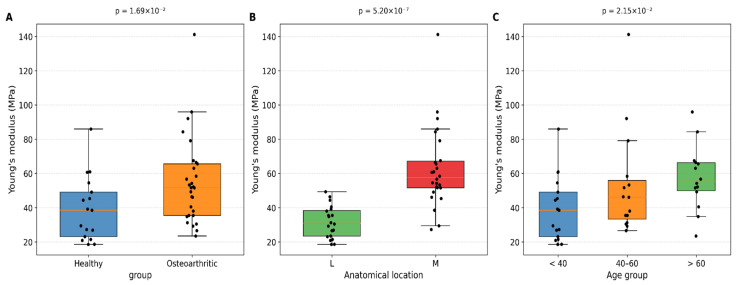
Comparative analysis of the Young’s modulus of articular cartilage across three key biomechanical determinants: (**A**) Boxplot comparing the elastic modulus between healthy and osteoarthritic samples, (**B**) Comparison of Young’s modulus between the medial and lateral compartments, (**C**) Relationship between donor age and cartilage stiffness.

Figure 4A illustrates the distribution of Young’s modulus between healthy and osteoarthritic samples. Figure 4B shows a substantial stiffness difference between medial and lateral compartments.

Figure 4C shows the distribution of Young’s modulus across three age groups: less than 40, 40–60, and greater than 60 years. The youngest group had the lowest median modulus values and the largest variability. The middle-aged group showed moderately higher median values, while the oldest group exhibited the highest median stiffness with the lowest variability.

### 3.4. Maximum Compressive Stress

Maximum compressive stress differed significantly according to both health status and anatomical location (Table 1). Osteoarthritic cartilage exhibited significantly higher maximum stress compared with healthy tissue.

**Figure 5:**
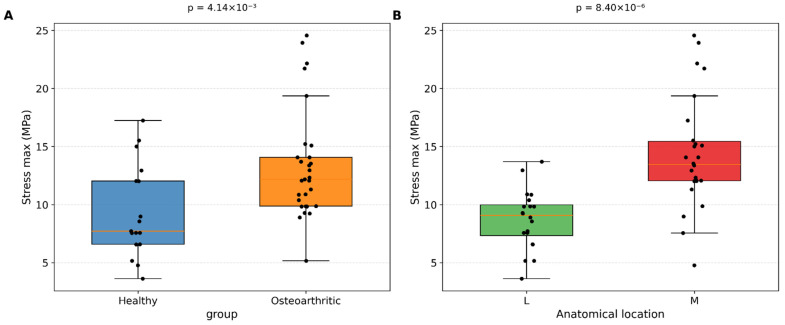
Comparative analysis of maximum compressive stress of articular cartilage: (**A**) Boxplot comparing maximum compressive stress between healthy and osteoarthritic samples, (**B**) Comparison of maximum compressive stress between the medial and lateral compartments.

Figure 5A illustrates the distribution of maximum stress values between healthy and osteoarthritic samples. Healthy specimens were primarily distributed within a lower and narrower stress range, whereas osteoarthritic cartilage showed a broader distribution with several high-value observations.

Figure 5B presents the anatomical comparison, showing that the medial compartment consistently exhibited higher maximum stress values than the lateral compartment.

### 3.5. Post Hoc Power Analysis

Post hoc power analysis (*α* = 0.05, two-tailed) demonstrated that the achieved sample sizes provided adequate statistical power for all primary comparisons. Power exceeded 80% for energy absorption (84%), Young’s modulus (97%), and maximum stress (>99%), confirming sufficient sensitivity to detect the observed effect sizes.

Repeatability assessment performed on a representative sample yielded a coefficient of variation of 18.2%, indicating acceptable measurement consistency.

### 3.6. Viscoelastic Model Performance

**Figure 6:**
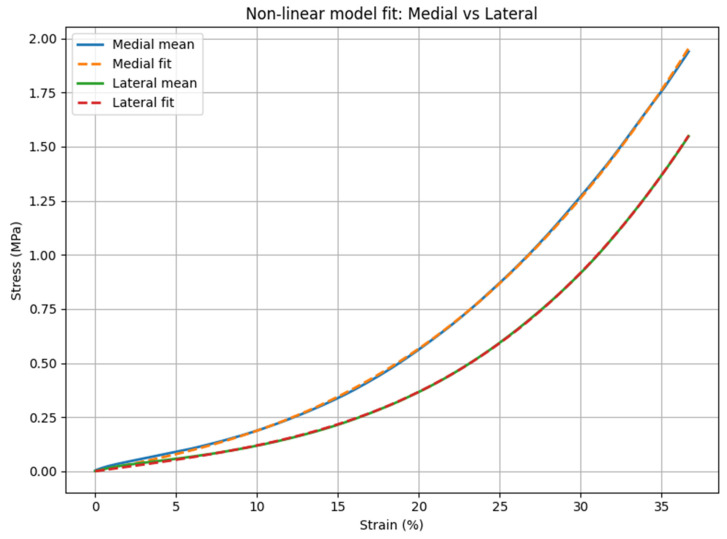
Fitted nonlinear phenomenological viscoelastic model overlaid on experimental mean curves for medial and lateral cartilage. The model closely follows the experimental response, reflecting an accurate reconstruction of nonlinear viscoelastic behavior.

The nonlinear phenomenological viscoelastic model was fitted to the experimental mean stress–strain curves for the lateral and medial cartilage compartments (Figure 6). The fitted curves followed the experimental data over the entire strain range for both compartments. For similar strain levels, higher stress values were observed in the medial compartment compared with the lateral compartment. The optimized model parameters are presented in Table 2. The medial compartment showed higher equilibrium modulus (*E∞* = 1.45 MPa), with viscoelastic coefficients *E*_1_ = 13.28 MPa and *E*_2_ = 8.07 MPa. The corresponding nonlinearity exponents were α_1_ ≈ α_2_ ≈ 2.70. The coefficient of determination was *R*^2^ = 0.99985. The lateral compartment exhibited *E∞* = 1.03 MPa, with *E*_1_ ≈ 0 and *E*_2_ = 31.20 MPa, and *α*_2_ ≈ 3.27. The coefficient of determination was *R*^2^ = 0.99995.

### 3.7. Model Sensitivity Analysis

**Figure 7:**
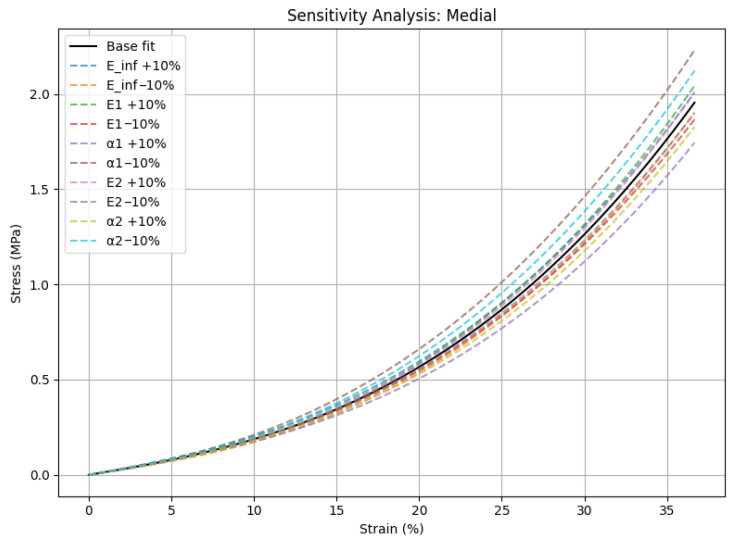
Sensitivity analysis of medial cartilage model parameters using ±10% perturbation. Minimal changes in *R*^2^ indicate high model robustness.

**Figure 8:**
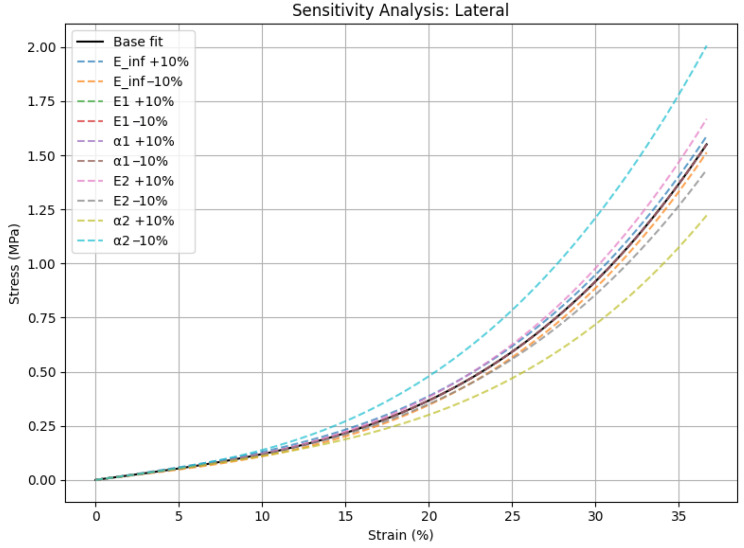
Sensitivity analysis of lateral cartilage model parameters with ±10% perturbation. Results confirm strong numerical stability and reduced dependence on secondary viscoelastic terms.

Sensitivity analysis was performed by applying ±10% perturbations to each model parameter, including the elastic moduli (*E∞*, *E*_1_, *E*_2_) and the nonlinearity exponents (*α*_1_, *α*_2_), resulting in only minimal changes in the coefficient of determination. Figure 7 presents the sensitivity analysis for the medial cartilage parameters, while Figure 8 shows the corresponding analysis for the lateral compartment. The *R*^2^ values remained extremely high, exceeding 0.99985 for the medial compartment and 0.99995 for the lateral compartment (Table 3).

## 4. Discussion

This study characterized the mechanical properties and viscoelastic behavior of healthy and osteoarthritic articular cartilage across medial and lateral tibiofemoral compartments. The main findings demonstrate that osteoarthritic cartilage exhibits significantly greater energy absorption via pathological deformation mechanisms, increased stiffness, and higher maximum stress than healthy tissue. Medial compartments consistently demonstrated greater mechanical resistance than lateral regions. The nonlinear phenomenological viscoelastic model accurately captured the tissue’s viscoelastic response, with an excellent fit (*R*^2^ > 0.999). Together, these findings provide quantitative evidence of biomechanical alterations associated with cartilage degeneration under conditions relevant to high-impact athletic activity.

### 4.1. Energy Absorption and Pathological Deformation

Osteoarthritic cartilage exhibited significantly higher energy absorption than healthy tissue, with a moderate-to-large effect size, indicating altered mechanical behaviours associated with degeneration. This increase does not reflect improved mechanical function but is instead related to matrix degradation and compositional changes characteristic of osteoarthritis [22]. Increased interstitial fluid mobility associated with elevated water content in degenerated tissue may further contribute to enhanced viscous energy dissipation [23]. When taken as a whole, these changes point to decreased structural integrity and heightened vulnerability to mechanical fatigue under conditions of repeated loading [24]. Furthermore, the energy absorption of the medial compartment was significantly greater than that of the lateral compartment, which is in line with the medial compartment’s primary load-bearing role during walking and sports [25].

### 4.2. Stiffness Alterations and Subchondral Influences

Osteoarthritic cartilage exhibited significantly higher Young’s modulus than healthy tissue, indicating reduced compliance and deformation capacity under compression. This apparent stiffening likely reflects structural changes associated with osteoarthritis, including subchondral bone sclerosis, increased trabecular density, and collagen crosslinking, which collectively influence the mechanical response of full-thickness cartilage samples [26,27]. Measurements integrate contributions from both superficial and deeper cartilage zones, which may mask regional softening while showing an overall increase in stiffness. Previous studies have reported both increased and decreased stiffness in osteoarthritic cartilage depending on disease stage, testing methodology, and anatomical location [28,29].

The medial compartment demonstrated markedly higher Young’s modulus than the lateral compartment, consistent with its primary load-bearing role during dynamic activities and anatomical specialization [30]. Computational models and experimental studies indicate that the medial compartment experiences peak stresses during valgus loading, cutting maneuvers, and single-leg support typical of football and pivoting sports [31]. The stiffer medial cartilage provides greater resistance to compressive forces but may be more vulnerable to cumulative microdamage under repetitive high-impact loads. These findings support clinical observations of medial-dominant cartilage lesions in athletes [32] and highlight the relevance of regional mechanical specialization in interpreting injury patterns in high-demand sports.

### 4.3. Maximum Stress and Tissue Failure Mechanisms

The maximum stress of osteoarthritic cartilage was significantly higher than that of healthy tissue, suggesting a change in the cartilage’s ability to withstand stress. Osteoarthritic cartilage may withstand high-magnitude loads despite structural degradation, although its efficiency and safety margins are diminished. Due to varying matrix degradation and localized structural weaknesses, the broad stress range (5–25 MPa) reflects heterogeneous mechanical properties [33]. During athletic activities, repeated exposure to high stresses may surpass tissue fatigue thresholds, encouraging the accumulation of microdamage and raising the risk of focal lesions.

The medial compartment sustained significantly higher maximum stress than the lateral compartment, consistent with its primary load-bearing function and elevated stiffness [34]. This mechanical asymmetry aligns with clinical and computational evidence showing that medial cartilage is more prone to focal lesions in football players, due to convergence of anatomical specialization, chronic high-magnitude loading, and sport-specific stress patterns.

### 4.4. Viscoelastic Modeling and Mechanical Signatures

The nonlinear phenomenological viscoelastic model successfully replicated the strain-dependent stiffening of articular cartilage under quasi-static compression, achieving high fit quality (*R*^2^ > 0.999) for both medial and lateral compartments. The model accurately represented the biphasic mechanical response, exhibiting quasi-linear behavior at low stresses and a noticeable stiffening at larger deformations. Two viscoelastic branches contributed equally to the medial cartilage (*E*_1_ = 13.28 MPa, *E*_2_ = 8.07 MPa; *α*_1_ ≈ *α*_2_ ≈ 2.70), indicating distributed energy dissipation over several relaxation time-scales. In contrast, lateral cartilage showed a dominant single branch (*E*_2_ = 31.20 MPa; *α*_2_ ≈ 3.27), indicating a more abrupt stiffening response, consistent with its lower in vivo mechanical demand.

Sensitivity analysis verified the robustness of the model by showing that the fitted parameters consistently reflect mechanical characteristics instead of overfitting artifacts, with very small changes in *R*^2^ after ±10% perturbations of each parameter. All things considered, these findings provide quantitative evidence of anatomical specialization in viscoelastic behavior and highlight the potential of mechanical signatures as early markers of osteoarthritic changes and tools for individualized injury-risk assessment in athletes [35].

### 4.5. Implications for Athletic Populations and Injury Prevention

The mechanical alterations documented in osteoarthritic samples provide essential insights into mechanisms of cartilage degeneration relevant to athletic populations exposed to repetitive high-impact loading. Football players’ cartilage structural integrity is challenged by rapid high-magnitude stress cycles and short recovery times. Osteoarthritic tissue’s increased stiffness and modified viscoelastic buffering are similar to the progressive mechanical changes that athletes’ joints may experience over their careers [36]. Longitudinal studies of former elite athletes demonstrate accelerated cartilage degeneration and earlier onset of osteoarthritis compared with non-athletic groups, emphasizing cumulative mechanical loading as a major risk factor [37]. Quantitative measurements of cartilage mechanical characteristics, including Young’s modulus, energy absorption, and viscoelastic relaxation parameters, may serve as sensitive biomarkers for detecting early degenerative alterations before morphological defects become apparent.

Clinical patterns of cartilage damage in football players are consistent with the anatomical vulnerability of the medial compartment. MRI studies report that lesions are predominantly located in medial weight-bearing regions, with prevalence exceeding 40% in certain cohorts despite young age [30,38]. This medial predominance may be attributed to greater baseline stiffness, increased stress exposure during sport-specific movements, and limited mechanical adaptability. These findings highlight the potential of load-redistribution strategies, including neuromuscular training and movement pattern modification, to reduce medial compartment stress and potentially delay cartilage degeneration in high-impact sporting populations.

In terms of future possibilities, one focus is on using artificial intelligence (AI) and wearable technology to improve the monitoring of athletes and help reduce their risk of injury. Through the use of AI motion analysis, it will be possible to develop an individualized assessment of the joint loading and movement patterns of each athlete, thus enabling the detection of biomechanical risk factors leading to cartilage damage and creating customized training interventions or methods that will facilitate athletes reaching their goals [39]. In addition, advanced AI techniques such as deep learning, which can analyze clinical data, have the ability to synthesize imaging data, biomechanical data, and clinical data in order to build predictive models for assessing the potential of injuries or maintaining long-term joint health for athletes competing at a high level in sports where they are subjected to high forces on their joints [40].

### 4.6. Limitations

Several methodological limitations warrant consideration when interpreting the present findings. First, unconfined compression testing was performed at room temperature (20 °C) rather than at physiological temperature (37 °C), which may influence the measured mechanical properties given the temperature sensitivity of cartilage viscoelastic behavior. Temperature affects interstitial fluid viscosity and matrix component interactions, potentially altering both stiffness measurements and viscoelastic parameters. Second, the quasi-static loading rate (1 mm/min) employed in this study is relatively slow compared with the rapid loading rates encountered during athletic activities. Cartilage exhibits strain-rate-dependent mechanical properties, with higher loading velocities generally producing increased apparent stiffness and altered energy dissipation patterns. The present findings, therefore, characterize the tissue’s equilibrium mechanical response rather than its behavior under dynamic impact conditions more representative of sports loading scenarios. Third, the nonlinear phenomenological viscoelastic model employed assumes homogeneous material properties throughout the sample thickness. In contrast, articular cartilage exhibits well-documented zonal variations in composition and mechanical properties from the superficial zone to the deep zone. This simplified modeling approach may not fully capture the depth-dependent mechanical behavior and could average across regional differences that influence overall tissue performance. Fourth, the cross-sectional study design precludes assessment of longitudinal changes in mechanical properties within individuals and direct causal inference regarding the progression from healthy to osteoarthritic states. Finally, the sample collection from surgical and post-mortem sources introduces potential selection bias, as tissue quality may differ from that of living athletic populations experiencing early degenerative changes.

## 5. Conclusions

This study provides a comprehensive mechanical characterization of human tibiofemoral cartilage using unconfined compression and nonlinear viscoelastic modelling. Osteoarthritic cartilage exhibits elevated energy absorption, increased stiffness (Young’s modulus), and higher maximum stress compared with healthy tissue, reflecting pathological mechanical remodelling rather than functional superiority. The medial compartment consistently demonstrates greater stiffness and stress than the lateral compartment, highlighting anatomical specialization and mechanical vulnerability.

The nonlinear phenomenological viscoelastic model accurately captured viscoelastic behaviour (*R*^2^ > 0.999), revealing distinct compartment-specific mechanical signatures. Medial cartilage displayed a balanced two-branch response, whereas lateral cartilage exhibited a dominant single-branch behaviour, providing quantitative parameters that may serve as early biomarkers of cartilage degeneration.

These findings have direct implications for athletes exposed to repetitive high-impact loading, such as football players, in whom medial cartilage appears particularly susceptible to injury and osteoarthritis. Quantitative assessment of cartilage mechanics may support early detection of degeneration, guide repair tissue evaluation, and inform load management and neuromuscular training strategies aimed at preserving joint health. Future research should investigate longitudinal mechanical changes and integrate subject-specific parameters into computational models for personalized injury risk assessment.

## Figures and Tables

**Table 1:** Descriptive and comparative statistics of cartilage mechanical properties according to tissue condition (Healthy vs. OA) and anatomical location (Lateral vs. Medial).

Parameter	Group	N	Mean ± SD	*p* Value	Effect Sizes
Energy	Healthy	17	1786.05 ± 1549.48	9.48 × 10^−3 a^	d = 0.61
	Osteoarthritic	29	2437.97 ± 1119.04		
	Lateral (L)	20	1759.08 ± 974.99	3.25 × 10^−2 a^	d = 0.66
	Medial (M)	26	2533.94 ± 1458.53		
Young’s Modulus (MPa)	Healthy	17	39.1 ± 14.96	1.69 × 10^−2 a^	d = 0.96
	Osteoarthritic	29	55.18 ± 18.15		
	Lateral (L)	20	31.71 ± 7.82	5.20 × 10^−7 b^	$r_{rb}\;$= 0.78
	Medial (M)	26	62.72 ± 16.45		
Maximum stress (MPa)	Healthy	17	9.37 ± 3.35	4.14 × 10^−3 a^	d = 1.16
	Osteoarthritic	29	13.37 ± 3.47		
	Lateral (L)	20	8.71 ± 1.99	8.40 × 10^−6 b^	$r_{rb}\;$= 0.67
	Medial (M)	26	14.34 ± 3.57		

^a^ Welch’s *t*-test (normal distribution, unequal variances): Energy (Healthy vs. OA), Energy (L vs. M), Young’s modulus (Healthy vs. OA), Maximum stress (Healthy vs. OA). ^b^ Mann–Whitney U test (non-normal distribution): Young’s modulus (L vs. M), Maximum stress (L vs. M). Kruskal–Wallis was used as an omnibus test before pairwise comparisons. Effect sizes were computed using Cohen’s d (parametric) or rank-biserial correlation $r_{rb}$ (non-parametric).

**Table 2:** Fitted model parameters (Non-linear Phenomenological Viscoelastic Model).

Anatomical Location	$E_{\infty }$ (MPa)	$E_{1}$ (MPa)	$\alpha_{1}$	$E_{2}$ (MPa)	$\alpha_{2}$	R^2^
Medial	1.4466	13.2848	2.6988	8.07213	2.698780	0.999850
Lateral	1.0250	0.000003	3.2848	31.1976	3.2683	0.999952

**Table 3:** Sensitive analysis summary.

Anatomical Location	Parameter	Variation Applied	R^2^
Medial	$E_{\infty }$ (MPa)	10%	0.999850
	$E_{1}$ (MPa)	10%	0.999850
	$\alpha_{1}$	10%	0.999850
	$E_{2}$ (MPa)	10%	0.999850
	$\alpha_{2}$	10%	0.999850
Lateral	$E_{\infty }$ (MPa)	10%	0.999952
	$E_{1}$ (MPa)	10%	0.999952
	$\alpha_{1}$	10%	0.999952
	$E_{2}$ (MPa)	10%	0.999952
	$\alpha_{2}$	10%	0.999952

## Data Availability

The data supporting the findings of this study are available from the corresponding authors upon reasonable request.

## References

[1] Belluzzi E., Todros S., Pozzuoli A., Ruggieri P., Carniel E.L., Berardo A. (2023). Human cartilage biomechanics: Experimental and theoretical approaches towards the identification of mechanical properties in healthy and osteoarthritic conditions. Processes.

[2] Horkay F., Basser P.J., Geissler E. (2024). Cartilage extracellular matrix polymers: Hierarchical structure, osmotic properties, and function. Soft Matter.

[3] Yue L., Lim R., Owens B.D. (2024). Latest advances in chondrocyte-based cartilage repair. Biomedicines.

[4] He J., Wang Z., Sun C., Li X., Zhang Y. (2025). Epidemiological trends of osteoarthritis at global, regional, and national levels from 1990 to 2021 and projections to 2050. Arthritis Res. Ther..

[5] Russell E.R., Spencer S.J., Atherton C.M., Lyall D.M., Mackay D.F., Stewart K., A MacLean J., Pell J.P., Stewart W. (2023). Increased risk of lower limb osteoarthritis among former professional soccer (football) players. Occup. Med..

[6] Petrigna L., Trovato B., Roggio F., Castorina A., Musumeci G. (2023). Molecular assessment of healthy and pathological articular cartilages in physically active people: A scoping review. Int. J. Mol. Sci..

[7] Verheul J., Harper D., Robinson M.A. (2024). Forces experienced at different levels of the musculoskeletal system during horizontal decelerations. J. Sports Sci..

[8] Collins J., Yang Y., Opare-Addo M., Losina E. (2023). Contribution of collagen degradation and proteoglycan depletion to cartilage degeneration in primary and secondary osteoarthritis: An in silico study. Osteoarthr. Cartil..

[9] Dalos D., Marshall P.R., Lissy M., Maas K.J., Henes F.O., Kaul M.G., Kleinertz H., Frings J., Krause M., Frosch K.H. (2024). Influence of leg axis alignment on MRI T2* mapping of the knee in young professional soccer players. BMC Musculoskelet. Disord..

[10] Bezuglov E.N., Lyubushkina A.V., Khaitin V.Y., Tokareva A.V., Goncharov E.N., Gorinov A.V., Sivakova E.Y., Sereda A.P. (2019). Prevalence of asymptomatic intra-articular changes of the knee in adult professional soccer players. Orthop. J. Sports Med..

[11] Linus A., Tanska P., Sarin J.K., Nippolainen E., Tiitu V., Mäkelä J.T.A., Töyräs J., Korhonen R.K., Mononen M.E., Afara I.O. (2024). Site-specific elastic and viscoelastic biomechanical properties of healthy and osteoarthritic human knee joint articular cartilage. J. Biomech..

[12] Alcaide Ruggiero L., Molina Hernández V., Granados M.M., Domínguez J.M. (2023). Proteoglycans in articular cartilage and their contribution to chondral injury and repair mechanisms. Int. J. Mol. Sci..

[13] Song J., Zeng X., Li C., Yin H., Mao S., Ren D. (2024). Alteration in cartilage matrix stiffness as an indicator and modulator of osteoarthritis. Biosci. Rep..

[14] Niasar E.H.A., Hamsayeh Abbasi N. (2025). Implication of region-dependent material properties of articular cartilage in the contact mechanics of the porcine knee joint. BMC Musculoskelet. Disord..

[15] Peters A.E., Akhtar R., Comerford E.J., Bates K.T. (2018). The effect of ageing and osteoarthritis on the mechanical properties of cartilage and bone in the human knee joint. Sci. Rep..

[16] Vazquez K.J., Andreae J.T., Henak C.R. (2019). Cartilage-on-cartilage cyclic loading induces mechanical and structural damage. J. Mech. Behav. Biomed. Mater..

[17] Crolla J.P., Lawless B.M., Cederlund A.A., Aspden R.M., Espino D.M. (2022). Analysis of hydration and subchondral bone density on the viscoelastic properties of bovine articular cartilage. BMC Musculoskelet. Disord..

[18] Mäkelä J.T.A., Korhonen R.K. (2016). Highly nonlinear stress-relaxation response of articular cartilage in indentation: Importance of collagen nonlinearity. J. Biomech..

[19] Xu Q., Engquist B., Solaimanian M., Yan K. (2020). A new nonlinear viscoelastic model and mathematical solution of solids for improving prediction accuracy. Sci. Rep..

[20] Dergaa I., Saad H.B., Glenn J.M., Aissa M.B., Taheri M., Swed S., Guelmami N., Chamari K. (2024). A thorough examination of ChatGPT-3.5 potential applications in medical writing: A preliminary study. Medicine.

[21] Dergaa I., Feikh-Romdhane F., Glenn J.M., Saifeddin Fessi M., Chamari K., Dhahbi W., Zghibi M., Bragazzi N.L., Ben Aissa M., Guelmami N. (2023). Moving beyond the stigma: Understanding and overcoming the resistance to the acceptance and adoption of artificial intelligence chatbots. New Asian J. Med..

[22] Khajehsaeid H., Abdollahpour Z., Farahmandpour H. (2021). Effect of degradation and osteoarthritis on the viscoelastic properties of human knee articular cartilage: An experimental study and constitutive modeling. Biomechanics.

[23] Wu Y., Ferguson S.J. (2017). The influence of cartilage surface topography on fluid flow in the intra-articular gap. Comput. Methods Biomech. Biomed. Eng..

[24] Zhang J., Lin W., Li J., Chen Z., Jin Z. (2025). Biomechanical effects of medial osteoarthritis progression and UKA on knee lateral compartment using fibril-reinforced biphasic material in finite element study. Sci. Rep..

[25] Maritz J., Agustoni G., Dragnevski K., Bordas S.P.A., Barrera O. (2021). The Functionally Grading Elastic and Viscoelastic Properties of the Body Region of the Knee Meniscus. Ann. Biomed. Eng..

[26] Savage M., Culvenor A.G., Hedger M., Matt A.R., O’Brien M.J.M., McMillan R.M., De Livera A., Mentiplay B.F. (2025). Are altered knee joint biomechanics associated with future post-traumatic osteoarthritis outcomes? A systematic review and meta-analysis of longitudinal studies. Sports Med..

[27] Huang K., Cai H. (2025). Matrix stiffness in osteoarthritis: From mechanism introduction to biomaterial-based therapies. Front. Endocrinol..

[28] Mieloch A.A., Richter M., Trzeciak T., Giersig M., Rybka J.D. (2019). Osteoarthritis severely decreases the elasticity and hardness of knee joint cartilage: A nanoindentation study. J. Clin. Med..

[29] Krakowski P., Rejniak A., Sobczyk J., Karpiński R. (2024). Cartilage integrity: A review of mechanical and frictional properties and repair approaches in osteoarthritis. Healthcare.

[30] Driban J.B., Hootman J.M., Sitler M.R., Harris K.P., Cattano N.M. (2017). Is participation in certain sports associated with knee osteoarthritis? A systematic review. J. Athl. Train..

[31] Zhang Y., Killen B.A., Mohout I., Arden N.K., Kluzek S., Zevenbergen L., van Middelkoop M., Runhaar J., Oei E.H.G., Jones G. (2025). Cartilage mechanical responses during gait as in silico biomarkers for medial knee OA progression. Sci. Rep..

[32] Li X., Kim J., Yang M., Ok A.H., Zbyn S., Link T.M., Majumdar S., Ma C.B., Spindler K.P., Winalski C.S. (2024). Cartilage compositional MRI—A narrative review of technical development and clinical applications over the past three decades. Skelet. Radiol..

[33] Sise C.V., Petersen C.A., Ashford A.K., Yun J., Zimmerman B.K., Vukelic S., Hung C.T., Ateshian G.A. (2025). A major functional role of synovial fluid is to reduce the rate of cartilage fatigue failure under cyclical compressive loading. Osteoarthr. Cartil..

[34] Feng R., Hu W., Li Y., Yao X., Li J., Li X., Zhang J., Wu Y., Kang F., Dong S. (2024). Mechanotransduction in subchondral bone microenvironment and targeted interventions for osteoarthritis. Mechanobiol. Med..

[35] de Roy L., Quelhas Teixeira G., Schwer J., Sukopp M., Faschingbauer M., Ignatius A., Seitz A.M. (2024). Structure–function of cartilage in osteoarthritis: An ex vivo correlation analysis between its structural, viscoelastic and frictional properties. Acta Biomater..

[36] Trovato B., Petrigna L., Sortino M., Roggio F., Musumeci G. (2023). The influence of different sports on cartilage adaptations: A systematic review. Heliyon.

[37] Pillay L., Janse van Rensburg D.C., Ramkilawon G., Maas M., Orhant E., Rantanen J., Salo J., Kerkhoffs G., Gouttebarge V. (2023). Determination of the prevalence of knee and hip clinical osteoarthritis in the active professional male footballer and its association with pain, function, injury and surgery. Sports.

[38] Welsch G.H., Marc Regier M., Frosch K.H., Pachowsky M.L., Henes F.O., Adam G., Maas K.J., Warncke M.L. (2025). Whole-Organ Magnetic Resonance Imaging Score (WORMS) of the Knee in Professional Soccer Players. Cartilage.

[39] Souaifi M., Dhahbi W., Jebabli N., Ceylan H.I., Boujabli M., Muntean R.I., Dergaa I. (2025). Artificial Intelligence in Sports Biomechanics: A Scoping Review on Wearable Technology, Motion Analysis, and Injury Prevention. Bioengineering.

[40] Abdaoui H., Barki C., Dergaa I., Tlili K., Ceylan H.I., Bragazzi N.L., de Giorgio A., Ben Salah R., Boussi Rahmouni H. (2026). Accurate Clinical Entity Recognition and Code Mapping of Anatomopathological Reports Using BioClinicalBERT Enhanced by Retrieval-Augmented Generation: A Hybrid Deep Learning Approach. Bioengineering.

